# Biomedical Exploitation of Chitin and Chitosan via Mechano-Chemical Disassembly, Electrospinning, Dissolution in Imidazolium Ionic Liquids, and Supercritical Drying

**DOI:** 10.3390/md9091510

**Published:** 2011-09-09

**Authors:** Riccardo A. A. Muzzarelli

**Affiliations:** University of Ancona, IT-60100 Ancona, Italy; E-Mail: Muzzarelli.raa@gmail.it; Tel./Fax: +39-071-36206

**Keywords:** chitin, chitosan, electrospinning, ionic liquids, nanofibrils, supercritical carbon dioxide

## Abstract

Recently developed technology permits to optimize simultaneously surface area, porosity, density, rigidity and surface morphology of chitin-derived materials of biomedical interest. Safe and ecofriendly disassembly of chitin has superseded the dangerous acid hydrolysis and provides higher yields and scaling-up possibilities: the chitosan nanofibrils are finding applications in reinforced bone scaffolds and composite dressings for dermal wounds. Electrospun chitosan nanofibers, in the form of biocompatible thin mats and non-wovens, are being actively studied: composites of gelatin + chitosan + polyurethane have been proposed for cardiac valves and for nerve conduits; fibers are also manufactured from electrospun particles that self-assemble during subsequent freeze-drying. Ionic liquids (salts of alkylated imidazolium) are suitable as non-aqueous solvents that permit desirable reactions to occur for drug delivery purposes. Gel drying with supercritical CO_2_ leads to structures most similar to the extracellular matrix, even when the chitosan is crosslinked, or in combination with metal oxides of interest in orthopedics.

## 1. Introduction

A large body of knowledge exists today on the use of chitosans as safe biomaterials for a variety of applications: there are recent review articles in biomedical sciences [1–6] and in pharmaceutical sciences [7–15]. Besides the review articles most directly dealing with chemical approaches [16–21], some are focused on the preparation and applications of carboxymethyl and succinyl derivatives of chitin and chitosan with particular attention to their biomedical applications [22]; on the hydrophobic modifications of chitosans mainly for gene delivery in comparison with polyethyleneimine and polylysine [23], while more general treatments are also available [24–26]. Reviews on the safety of chitin have been published recently [27,28].

Chitins and chitosans and their beneficial characteristic properties could certainly be even further improved by more elaborate refinements of the technological approaches to enable further exploitation of the amply available chitin resources. The scope of the present review article is to provide technical details and evaluations of results, suitable for the appreciation of the immediate potential developments of the title technologies.

## 2. Chitin and Chitosan Nanofibrils

In the area of the isolation and characterization of nanofibrils (otherwise called chitin nanocrystals, or whiskers), significant scientific and technological advances have recently been made. In particular, articles were published dealing with: (i) disassembly of chitin for the isolation of nanochitin by mechanical means in the presence of minor amounts of acetic acid; (ii) disassembly of chitosan; (iii) preparation of nanochitosan from partially deacetylated chitin.

### 2.1. Mechanical Disassembly of Chitin Nanofibrils

Chitin nanofibers were prepared by Kose and Kondo [29] by using the aqueous counter collision method that provided homogeneous aqueous dispersion of chitin nanofibers having a width of 10–20 nm. The mechanical disassembly of chitin has suddenly attracted much attention: in fact, suspensions of crude α-chitin were treated first with a blender, and then the slurry of 1% purified chitin was passed through a grinder at 1500 rpm, with a clearance gauge of 0.15 mm shift as demonstrated by Ifuku *et al.* [30].

The same team developed the concept that it would be advantageous to enhance the cationic repulsion existing between chitin fibers with the aid of partial protonation in order to disassemble the chitin: a protonation degree as small as 4% or less is sufficient to weaken the hydrogen bonds that protect the tight chitin structure [31]. Various industrial chitins, including the α-polymorphs, by this route yield nanofibrils having a high degree of crystallinity and 10–20 nm cross-section.

According to Shams *et al.* [32] several drops of acetic acid were added to the 1% slurry of purified wet chitin to adjust the pH value at 3–4 and to facilitate the fibrillation: the suspension was then blended for 10 min at a speed of 37,000 rpm, and kept in a never-dried condition. The neutralized disassembled nanofibrils were dispersed in water (0.1%) and a colloidal structure was obtained, indicating that the chitin fibrils were homogeneously dispersed: it was vacuum-filtered on a polytetrafluoroethylene membrane (0.1 μm porosity) to produce a dry sheet having diameter 9 cm, thickness 55 μm, and density 1.0 g/cm^3^. The dried sheets were impregnated with neat acrylic resin (refractive index 1.536) in such a way as to obtain transparent nanocomposites, with 40% chitin content. The width distribution evaluated directly from the SEM images demonstrated that almost 70% of the nanofibril width was in the range 20–30 nm. The degree of *N*-acetylation 0.93 of the nanofibers was obtained by elemental analysis and indicated that no deacetylation occurred in the course of the treatment. Furthermore, the polysaccharide did not lose its transparency because fibers or particles which have such a small diameter do not produce light scattering; as a consequence it was claimed useful for optical devices. As an extension of that work, optically transparent chitin nanofibril composites were fabricated with 11 different types of (meth)acrylic resins. Chitin nanofibers significantly increased the Young’s modulus and the tensile strength, and decreased the thermal expansion of all (meth)acrylic resins due to the reinforcement effect of chitin nanofibers endowed with extended crystal structure [33,34].

Incidentally, under the same conditions, ultrasonication was applied to the chitin slurries for 2 min using an ultrasonic homogenizer at 19.5 kHz and the 300 W output power (probe tip diameter 7 mm); the temperature increase was <5 °C during the ultrasonication [35].

The disadvantages of the earlier preparation methods were numerous and included low yield, dangerous handling of boiling HCl, disposal of the colored HCl solution, recovery of enormous quantities of slightly acidic water, difficult adjustment of the pH value because of the strength of HCl, scaling-up troubles and excessive costs. The new technology that permits today the treatment of industrial chitin dry powders instead of “never dried” chitins removes the most important limitation of the large-scale production of nanochitin. This technology provides a significant advantage towards chitin exploitation, in terms of transportation costs, stable supply, shelf life and storage space, since chitin nanofibers can be prepared from light, low volume, and non-perishable dry chitin.

### 2.2. Mechanical Disassembly of Chitosan Nanofibrils

High-pressure homogenization was combined with wet-grinding to disassemble suspended chitosan particles into nano-chitosan [36]. The chitosan slurry was forced to pass through the wet-grinding machine at the flow rate of 10 L/h and then it was poured into the stainless-steel tank of a Microfluidizer (M-100P, Microfluidics Corp. MA, USA) equipped with a pair of ceramic (200 μm) and diamond (87 μm) interaction chambers; it was cooled and then released back to the tank for the next cycle. Under the pressure of 207 MPa, the ground slurry passed 10 times through the interaction chambers at the flow rate of 8 L/h; then, the homogenized chitosan slurry was centrifuged at 1000 rpm for 5 min to remove the sediment and to yield a homogeneous chitosan suspension that was used to prepare a high strength liquid crystal thin film at relatively low temperature. There was no pattern or fingerprint found in the control cast films, meaning that chitosan nanofibrils self-assembled with cholesteric structure. Whilst the chitosan cast film possessed high tensile strength (about 35.8 ± 7.6 MPa) and Young’s modulus (about 580.0 ± 21.8 MPa), the chitosan liquid crystal film had higher values up to 100.5 ± 4.0 MPa, and 2.2 ± 0.2 GPa, respectively, that are typical for liquid crystalline polymers [37].

### 2.3. Nanochitosan Obtained from Partially Deacetylated Chitin, or from Deacetylated Nanochitin

For this preparation, the deacetylation of chitin nanofibrils was made with 50% NaOH in the presence of borohydride; the molecular weight dropped to 59 kDa, much lower than the one of chitosan from chitin powder under the same conditions (422 kDa) [38]. The degree of deacetylation was 0.50 and the suspensions were colloidal at 1–13%. The new methods however opened new routes directly to nanofibrillar chitosan, which is more versatile than chitin. The fine chitin powder was also deacetylated in a relatively mild way, thus producing nano-chitosan that underwent homogeneous dispersion at pH 3–4 [39].

### 2.4. Applications

From the cell walls of five different types of mushrooms, chitin nanofibrils were isolated by removing glucans, minerals, and proteins, and subsequent grinding treatment under acidic conditions as described above. The chitin nanofibrils thus obtained by Ifuku *et al.* [40] were characterized by elemental analysis, FTIR spectrometry, and X-ray diffraction; they had uniform structure and were unusually long. The width of the nanofibers was in the range 20–28 nm and depended on the type of mushroom. The results showed that the α-chitin structure was maintained and glucans remained on the nanofiber surface. It was deemed that the said nanofibrils of fungal origin might have anti-tumor applications and immune-modulating activity.

**Figure 1:**
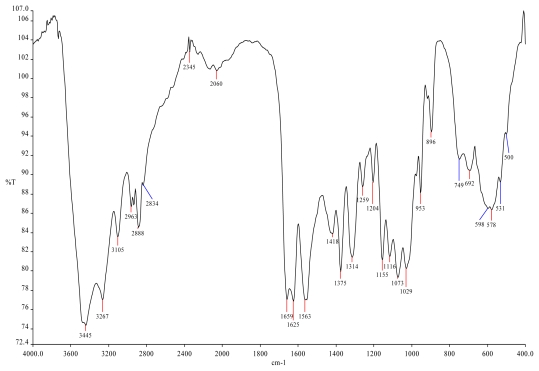
FTIR spectrum of spray-dried α-chitin nanofibrils ready for incorporation in a chitin + chitosan composite used for wound dressing. This spectrum showed for the first time unmatched resolution of all typical chitin bands. Reprinted from [41]. Copyright (2007) with permission from Elsevier.

Exhaustive studies were made by Muzzarelli *et al.* [41] who incorporated crustacean chitin nanofibrils into wound dressings made of chitosan glycolate and dibutyryl chitin that were applied in a variety of traumatic wounds with limited number of changes and excellent final healing; the nanofibrils were characterized with advanced instrumental analytical techniques (Figure 1).

Han *et al.* [42] investigated the influence of chitosan nanofiber scaffold on the production and infectivity of porcine endogenous retrovirus expressed by porcine hepatocytes. Freshly isolated porcine hepatocytes were cultured with a chitosan nanofiber scaffold, that prolonged the porcine endogenous retrovirus secreting time in pig hepatocytes, but did not appreciably influence its productive amount and infectivity, so it could be applied in the bioartificial liver without risk of virus transmission.

Chitin nanofibrils 5–10 nm diameter were employed by Ma *et al.* [43] as barrier layers in a new class of thin-film nanofibrous composite membranes for water purification. The very high surface-to-volume ratio leads to high virus adsorption capacity as verified by MS2 bacteriophage testing, and offers further opportunities in drinking water applications. The low cost of raw chitin, the environmentally friendly fabrication process, and the impressive high flux indicate that such ultrafine nanofibril-based membranes can surpass conventional-membranes in many water applications.

The chitin nanofibrils were effective in stabilizing oil-in-water emulsions against coalescence, presumably because of the adsorption of the nanofibrils at the oil–water interface. The rheological data provided evidence for network formation in the emulsions with increasing chitin nanocrystal concentration. Such a gel-like behavior was attributed to the formation of a chitin nanocrystal network in the continuous phase. The stability of the emulsions to creaming increased linearly with nanofibril concentration [44].

Several more applications of chitin nanofibrils have already been developed, for example, waterborne polyurethane-based nanocomposites were prepared by Huang *et al.* [45] by incorporating small quantities of chitin nanofibrils as the nanophase: the nanofibrils loading of 3% showed the maximum tensile strength (28.8 MPa) and enhanced the Young’s modulus (6.5 MPa), ~1.8- and 2.2-fold over those of neat polyurethane. The active surface and rigidity of nanofibrils facilitated formation of the interface for stress transferring and provided endurance to stress. The incorporation of chitosan nanofibrils in alginate fibers was achieved by mixing homogenized chitosan nanofibrils colloidal suspension with 6% w/v sodium alginate aqueous solution, followed by wet spinning [38].

Porous bone scaffolds with enhanced physical, mechanical and biological performances were prepared with hyaluronan and gelatin (1:1 w/w blend), and the reinforcing filler was α-nanochitin; 1-ethyl-3-(3-dimethylaminopropyl) carbodiimide was used as a crosslinker [46]. The weight ratios of the nanochitin to the blend were up to 30%, the average pore size of the scaffolds ranged between 139 and 166 μm, regardless of the nanochitin content, but the incorporation of 2% nanochitin in the scaffolds doubled their tensile strength. The as-prepared nanochitin was in the form of slender rods with sharp ends (255 ± 56 × 31 ± 6 nm, with L/d aspect ratio ~8). Although the addition of 20–30% nanochitin improved thermal stability and resistance to biodegradation, the scaffolds with 10% were the best for supporting the proliferation of cultured human osteosarcoma cells.

Melatonin was adsorbed on the nanofibrils [47,48]; lipoic acid was likewise treated [49]. Glycerol plasticized-potato starch was mixed with chitin nanofibrils to prepare fully natural nano-composites by casting and evaporation: this led to improvements in tensile strength, storage modulus, glass transition temperature, and water vapor barrier properties of the composite. However, at >5% loading, aggregation of the nanofibrils took place with negative effects [50]. On the other hand, the effect of different concentrations of cellulose nanofibers, and plasticizer (glycerol) on tensile properties, water vapor permeability, and glass transition temperature of chitosan edible films were evaluated to work out a formulation that optimized their properties: the nanocomposite film with 15% cellulose nanofibers and 18% glycerol, comparable to some synthetic polymers in terms of strength and stiffness [51].

Chitin nanofibrils were acetylated to modify the fiber surface: the acetylation degree could be controlled from 0.99 to 2.96 by changing the reaction time. After a short acetylation (1 min), the moisture content of the nanocomposite decreased from 4.0 to 2.2%. The nanofibril shape was maintained and the thickness of the nanofibrils increased linearly with the acetylation degree. Composites containing the acetylated chitin nanofibrils (25%) in acrylic resin were fabricated [34]. Nanofibers based on poly(vinyl alcohol) as the matrix, and α-chitin nanofibrils (~31 nm × ~549 nm) were prepared [52]. Chitin nanofibrils were blended with polylactide to form organic composites, and with apatite or rectorite to form inorganic composites suitable for food-packaging applications [53–55].

## 3. Electrospun Nanofibers

Electrospun natural biopolymers are of great interest in the field of regenerative medicine due to their unique structure, biocompatibility, and potential to support controlled release of bioactive agents and/or the growth of cells near a site of interest. However, the scaling-up of chitosan nanofiber fabrication by electrospinning is problematic and challenging. First of all, solutions with a high concentration of chitosan are not injectable, while those with a very low concentration result in a low output rate. On the other hand, the large quantity of organic solvents used during the electrospinning process alters/denatures the structure and properties of the natural chitosan. Furthermore, due to its polyelectrolyte nature, chitosan cannot be continuously spun as droplets persistently form. It is well known that pore size and structure of a scaffold play a vital role in cell cultures because they are responsible not only for the adhesion, migration, and distribution of cells, but also for the exchange of nutrients and metabolic waste. Despite numerous efforts, issues relating to mechanical strength, uniformity, interconnections and porosity of nanofiber mats have not yet been solved.

### 3.1. Chitosan + Nylon Electrospun Nanofibers

The electrospun chitosan has been characterized by Nirmala *et al.* [56] with the aid of the most advanced analytical instrumentation: they found that the chitosan nanofibers had diameters ranging from 10 to 1200 nm, and anisotropic nature. Their stability was studied by Cooper *et al.* [57], while Jacobs *et al.* [58] optimized the electrospinning parameters for chitosan nanofibers.

Electrospinning offers unique possibilities when a single compound dissolves both chitosan and an artificial polymer, this being the case for chitosan and nylon, so that the biochemical properties of chitosan can be associated to the mechanical properties of nylon: outstanding performances can be expected from such a composite, as mentioned by Muzzarelli [59] for the recovery of metals from sea-water. A work by Nirmala *et al.* [60] focused on the preparation of chitosan blended polyamide-6 nanofibers by a single solvent via electrospinning, to be used for cultures of human osteoblasts. The nanofibers were well oriented and had good incorporation of chitosan. Infrared spectrometry indicated that the amino groups of chitosan still existed in the blended nanofibers. The morphological features of the cells attached to nanofibers were observed by SEM. The adhesion, viability and proliferation properties of osteoblast cells on the polyamide-6 + chitosan blended nanofibers were analyzed by *in vitro* cell compatibility test. In a further work, Nirmala *et al.* [61] reported that current-voltage measurements revealed interesting linear relation, including enhanced conductivity with respect to chitosan content. The electrical conductivity of the polyamide-6 + chitosan composite nanofibers increased with increasing content of chitosan due to the formation of ultrafine nanofibers. In addition, the sheet resistance of composite nanofibers decreased with increasing chitosan concentration.

A totally different approach was followed by Zhang *et al.* [62]: electrospun nylon-6 nanofibrous membrane with fiber diameters in the range of 50–200 nm were prepared and employed as affinity material for papain adsorption due to their excellent chemical and thermal resistance as well as high wettability. Covalent coupling of chitosan to activated nylon membrane was performed after the reaction of the nanofibrous nylon membrane with formaldehyde. The dye Cibacron Blue F3GA as a ligand was then covalently immobilized on the chitosan-coated membranes, to be used for papain collection, with adsorption capacity up to 133.2 mg/g.

### 3.2. Applications in Cardiology

Hussain *et al.* [63] explored electrospun chitosan-based nanofiber scaffolds for cardiac tissue engineering. In the 2D and 3D scaffolds, only the cardiomyocyte-fibroblasts co-cultures resulted in polarized cardiomyocyte morphology, and the expression of sarcomeric actin and connexin-43 was higher than under other culture conditions. Said fibroblasts co-cultures demonstrated synchronized contractions involving large tissue-like cellular networks. This was the first attempt to utilize 3D chitosan nanofibers as cardiomyocyte scaffolds with the result that cardiomyocyte retained their morphology and function. Other applications in cardiology were proposed by Cynthia *et al.* [64] who tested mechanical properties, biocompatibility and cell retention ability of gelatin-chitosan polyurethane. In fact, for a proper function of the cardiac valve the materials selected for the leaflets need to be biocompatible, robust, flexible, and have comparable mechanical properties to the natural ones. Native heart valve leaflets are subjected to continuous pulsatile and homodynamic forces and can be as thin as 300 μm. Endothelial cells, isolated from ovine carotid arteries were seeded onto these materials and exposed to a range of shear-stresses for a period of 1–3 h. Throughout the exposure time and the shear stress values tested, a mean cell retention of 80% was obtained in the gelatin-chitosan polyurethane group. Noticeably for the full range of physiological flow conditions tested, the electrospun gelatin-chitosan polyurethane demonstrated good biocompatibility and cell retention properties.

Likewise, scaffolds for blood vessel and nerve conduits, were designed on the basis of collagen-chitosan-thermoplastic polyurethane electrospun to mimic the components and the structural aspects of the native extracellular matrix. The scaffolds were crosslinked with glutaraldehyde vapor to prevent them from being dissolved in the culture medium. Cell viability studies with endothelial cells and Schwann cells demonstrated that the electrospun composite nanofibrous scaffolds had good biocompatibility, and that the aligned fibers could regulate cell morphology by inducing cell orientation. Vascular grafts and nerve conduits were electrospun or sutured based on the nanofibrous scaffolds: the results indicated that collagen-chitosan-polyurethane blended nanofibrous scaffolds might be suitable for vascular repair and nerve regeneration [45].

### 3.3. Other Preparations of Biomedical Interest

An alternative to the experiments described above is the esterification of chitosan with the use of lactide or polylactide: both chitosan and polylactide/polyglycolide have good biocompatibility and can be used to produce scaffolds for cultured cells. However the synthetic scaffolds lack groups that would facilitate their modification, whereas chitosan has extensive active amine and hydroxyl groups which would allow subsequent modification intended for the attachment of peptides, proteins and drugs. Moreover chitosan is very hydrophilic, whereas poly(d,l-lactide-co-glycolide) (PLGA) is relatively hydrophobic. Accordingly there are many situations where it would be ideal to have a copolymer of both, especially one that could be electrospun to provide a versatile range of scaffolds for tissue engineering. In a study by Xie *et al.* [65], chitosan was first modified with trimethylsilyl chloride, in the presence of dimethylamino pyridine. PLGA-grafted chitosan copolymers were prepared by reaction with end-carboxyl PLGA (PLGA-COOH). Elemental analysis showed segments with an average of 18-pyranose units when PLGA-COOH was grafted into the chitosan chain. Contact angle measurements demonstrated that copolymers became more hydrophilic than PLGA. The chitosan-g-PLGA copolymers were electrospun to produce either nano- or microfibers as desired. A 3D fibrous scaffold of the copolymers gave good fibroblast adhesion and proliferation which did not differ significantly from the performance of the cells on the chitosan or PLGA electrospun scaffolds.

Chitosan derivatives were prepared by Skotak *et al.* [66] following a “one pot” approach by grafting l-lactide oligomers. Chitosan was dissolved in methanesulfonic acid, followed by the addition of the l-lactide monomer. This reaction mixture was stirred for 4 h at 40 °C under an argon atmosphere. The side chain had values between 4.6 and 14 units. On average, there were two side chains of oligo- l-lactide per glucosamine ring, and their length depended on the initial reagents ratio. l-Lactide grafted chitosan samples display cytotoxicity over a range of substitution degree values, as demonstrated with fibroblast cultures. This synthetic route renders the esterified chitosans soluble in a broad range of organic solvents, facilitating formation of ultrafine fibers via electrospinning.

Likewise, from blended solutions of chitosan and poly(ethylene oxide) (PEO), chitosan-based defect-free nanofibers with average diameters from 62 ± 9 nm to 129 ± 16 nm were fabricated [67]. The use of polysorbate surfactant Tween 20 to improve the functionality of the nanofibers was studied by Ziani *et al.* [68] with two highly deacetylated chitosans (MW 148 and 68 kDa) dissolved with acetic acid and then mixed with PEO. Because pure chitosan dissolved in different acid concentrations did not form fibers but beads, addition of PEO was necessary to electrospin the chitosan solutions. Average fiber diameters and size distribution depended on acidity and molecular weight. Solutions of chitosan + PEO + surfactant were effectively electrospun. The presence of surfactant resulted in decrease of surface tension and in the formation of smooth fibers.

Cationic nanofibrous mats are expected to show improved cellular adhesion and stability. Caprolactone oligomers were grafted onto the hydroxyl groups of chitosan via ring-opening polymerization by using methanesulfonic acid as solvent and catalyst: then, nanofibrous mats were obtained by electrospinning. The content of amino groups on the nanofiber surface increased linearly with the quantity of grafted chitosan, as revealed by the increased zeta-potential of nanofibers. Chitosan-*g*-oligo(caprolactone) in poly(caprolactone) (2/8) mats with moderate surface zeta-potential (3 mV) were the best in promoting mouse fibroblast attachment and proliferation. Toluidine blue staining confirmed that said cells grew well and exhibited a normal morphology [69]. Chitosan/poly(caprolactone) nanofibrous scaffold was prepared in a single step by Shalumon *et al.* [70]. The presence of chitosan in the scaffold imparted improved hydrophilicity to the scaffold, as confirmed by decreased contact angle, which thereby enhanced bioactivity and protein adsorption on the scaffold, which was found to be cyto-compatible.

Feng *et al.* [71] developed a method to obtain high surface area nanofiber meshes composed of chitosan of various molecular weights. These chitosan nanofiber meshes were developed as a culture substrate for hepatocytes: they exhibited a uniform diameter distribution (average 112 nm) and stability. The chitosan nanofibers were biocompatible with hepatocytes and were expected to be useful for artificial liver and for liver regeneration.

Aligned and randomly oriented poly(d, l-lactide-co-glycolide) + chitosan nanofibrous scaffolds have been prepared by electrospinning [72]. The release of the drug fenbufen from the fenbufen-loaded aligned and randomly oriented PLGA and PLGA/chitosan nanofibrous scaffolds increased with the increase of chitosan content. Moreover, the nanofiber arrangement influenced the release behavior. Crosslinking in glutaraldehyde vapor contributed to decrease the burst release of the drug from the loaded PLGA/chitosan nanofibrous scaffold.

Gelatin and chitosan nanofibers were electrospun and then cross-linked by glutaraldehyde vapor at room temperature. The cross-linked mats kept their nanofibrous structure after being soaked in deionized water at 37 °C. The two main chemical reactions of cross-linking for chitosan and gelatin-chitosan complex are Schiff base reaction and acetalization. The mechanical properties of nanofibrous mats were improved after cross-linking. The biocompatibility of electrospun nanofibrous mats after cross-linking was investigated by the viability of porcine iliac endothelial cells [73].

Silk fibroin + hydroxybutyl chitosan nanofibrous scaffolds were fabricated by electrospinning by using hexafluoro-2-propanol and trifluoroacetic acid as solvents to mimic the extra-cellular matrix. Both tensile strength and elongation at break were remarkably improved when the weight ratio of fibroin to hydroxybutyl chitosan was 20:80. The use of genipin vapor not only induced conformation of fibroin to convert from random coil to beta-sheet structure but also acted as a cross-linking agent for fibroin + hydroxybutyl chitosan [74].

As an example of versatility of the chitosan derivatives, a new method was presented by Almodovar and Kipper [75] for functionalizing electrospun nanofibers with GAGs and growth factors by polyelectrolyte multilayers deposition. Chitosan nanofibers, electrospun from trifluoroacetic acid and dichloromethane, were coated with heparin and N,N,N-trimethyl chitosan. FGF-2 was adsorbed on the PEM-coated nanofibers.

Electrospun nanofibers can be mineralized. The chitosan/poly(vinyl alcohol) nanofibers produced thin CaCO_3_ crystals that interlaced with the chitosan fiber not only on the surface of the membrane but also within it. The crystals developed into a continuous CaCO_3_ membrane on the fibers at a late stage of mineralization: the crystals were mainly calcite with a small quantity of vaterite. The attachment and growth of mouse fibroblast occurred evenly on the surface of the mineralized composite membrane [76].

Espindola-Gonzalez *et al.* [77] studied structural and thermal properties of chitosan + starch + poly(ethylene terephthalate) (PET) fibers manufactured via electrospinning. Addition of PET to chitosan + starch systems resulted in improved thermal stability at elevated temperatures.

**Figure 2:**
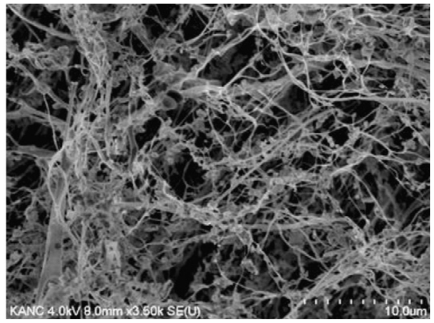
SEM micrograph of chitosan nonwoven fabrics obtained by electrospraying and subsequent freeze drying. Reprinted from [78]. Copyright (2011) with permission from Elsevier.

Kim and Lee [78] reported that the combination of electrospraying and subsequent freeze-drying can produce chitosan fibrous 3D network structures from low concentration chitosan solutions, well below fiber forming concentrations. Nanoparticle suspensions of chitosan were first fabricated by a controlled electrospraying process, and then the freeze-drying process promoted the assembly of the nanoparticles into fibrous networks. Figure 2 shows the typical fibrous structures obtained by electrospraying and subsequent freeze drying: the average diameter of a strand was 0.5–3 μm and the surface area of the fibers was 17.8 ± 0.39 m^2^/g. The nanofibrils, showing interconnections with each other were exempt from the bead-string motifs often found in electrospun fibers. The surface of these nanofibrils is different from those of conventional fibers insofar as it has no significant textures resulting from stretching processes, as a point of difference from the surface of most chitosan fibers.

Chitosan, sodium chondroitin sulfate, and pectin-nanofibrous mats were prepared from the respective polysaccharide/poly(ethylene oxide) blend solutions by electrospray. Unblended polysaccharide solutions showed low processability; viz., the solutions could not be electrosprayed. The addition of 500 kDa poly(ethylene oxide) to chitosan solutions enhanced the formation of a fibrous structure. Sodium chondroitin sulfate/poly(ethylene oxide) and pectin/poly(ethylene oxide) blend solutions were generally too viscous to be sprayed at 25 °C, but at 70 °C the fibrous structure was formed [79].

Chitosan fibers showing narrow diameter distribution with a mean of 42 nm were produced by electrospinning and utilized for the sorption of Fe(III), Cu(II), Ag(I), and Cd(II) ions from aqueous solutions. By virtue of its mechanical integrity, the applicability of the chitosan mat in solid phase extraction under continuous flow looks interesting: the surface area calculated from the isotherms to be about 0.92 m^2^/g for the powder and 22.4 m^2^/g for the electrospun fibers, indicates that electrospinning introduced a 20-fold increase of the surface area of chitosan [80]. Of course this kind of data on the chelating capacity of nanofibrous mats endowed with large surface area can be extended to the enhancement of the antibacterial activity of silver chelates of this kind. In fact, electrospun chitosan + poly(vinyl alcohol) nanofibers functionalized with silver nanoparticles had 220–650 nm diameter, and the silver nanoparticles embedded into the fibers exerted high antibacterial activity against *E. coli* [81]. Data assessing the doping effects of monovalent, bivalent and trivalent metal ions on the morphological appearance of the electrospun chitosan + poly(ethylene oxide) blend nanofibers were made available by Su *et al.* [82].

Chitosan nano-powders were modified using hydrazine plasma produced at low pressure (26.66 Pa) with 13.56 MHz frequency at 100 W for 30 min. Chitosan and plasma-modified chitosan in poly(vinyl alcohol) (PVA) solutions were used to produce nanofibers by electrospinning, with average fiber diameters 480 and 280 nm, respectively. The antibacterial effect of the treated chitosan was enhanced [83]. The spinnability of chitosan with PVA was optimized by Huang *et al.* [84].

## 4. Ionic Liquids: New Reaction Media

The ionic liquids have emerged as a class of organic salts that can be used as components of polymeric matrices, templates for porous polymers and solvents for a wide variety of organic and inorganic compounds. They are liquids at room temperature and exhibit unique physico-chemical properties, namely no vapor pressure, excellent chemical and thermal stability, high ionic conductivity and easy recyclability. Their use as solvents or non-volatile reaction media instead of conventional organic solvents can minimize a number of environmental and safety problems. They have provided a new processing platform for the dissolution of cellulose, chitin, starch and lignin: the macromolecules can be dissolved, regenerated and functionalized, thus increasing their chances of exploitation.

Not so much information on the dissolution of chitin in ionic liquids is present in the literature yet, because attention was immediately captured by cellulose that is dissolved likewise: typical ionic liquids in this context are 1-allyl-3-methylimidazolium chloride (AmiCl); 1-butyl-3-methylimidazolium chloride (BmiCl), and 1-butyl-3-methylimidazolium acetate (BmiAc) [85].

Xie *et al.* [86] used BmiCl and declared that up to 10% of chitin could be dissolved within 5 h at 110 °C; however, this finding was questioned because results were not reciprocally comparable, presumably due to the diversity of chitin in terms of polymorphic form, different origin, molecular weight and degree of acetylation.

An acidic cellulose + chitin gel electrolyte made of cellulose, chitin, 1-butyl-3-methylimidazolium, 1-allyl-3-methylimidazolium bromide, and an aqueous H_2_SO_4_ solution was investigated for electric double layer capacitors with activated carbon fiber cloth electrodes. The acidic cellulose + chitin hybrid gel electrolyte has practical applicability to an advanced electric double-layer capacitor with excellent stability and working performance [87]. Rheological evaluations on the clear solution of 5% chitin in 1-allyl-3-methylimidazolium bromide (obtained at 100 °C for 48 h) showed that it behaved like weak gels [88]. Chitosan + cellulose composite fibers from an ionic liquid medium were obtained by electrospinning [89].

Another example was provided by Takegawa *et al.* [90] who prepared chitin + cellulose composite gels and films using the two ionic liquids, 1-allyl-3-methylimidazolium bromide and 1-butyl-3-methylimidazolium chloride. Both polysaccharides were dissolved separately and then the two liquids were mixed in various ratios at 100 °C to give homogeneous mixtures: gels were obtained after 4 days. Moreover, films made of chitin + cellulose were obtained by casting the mixtures on glass plates, followed by soaking in water and drying: the obtained gels and films were characterized by X-ray diffraction spectrometry and thermo-gravimetric analysis. The mechanical properties of the gels and films were evaluated under compressive and tensile modes, respectively [90]. More data by Wu *et al.* [91] and by Kadokawa *et al.* [92] relevant to the solubility of chitins and chitosans in said ionic liquids are collected in Table 1.

The dissolution behavior of chitin in a series of ionic liquids containing alkylimidazolium chloride, alkylimidazolium dimethyl phosphate, and 1-allyl-3-methyl-imidazolium acetate has been studied by Wang *et al.* [93]. The dissolution behavior of chitin in ionic liquids was affected by the degree of acetylation, the degree of crystallinity, and the molecular weight of chitin, as well as by the nature of the anion of the ionic liquid. Moreover, 1-ethyl-3-methyl-imidazolium acetate dissolves raw crustacean shells completely, leading to the recovery of highly pure chitin of high molecular weight, in the form of powder, films and fibers directly spinnable from the extract solution [94].

Polyacrylamide was used by Zhou and Wu [95] as a matrix material for fabricating novel nanocomposite hydrogels reinforced with natural chitosan nanofibers via *in situ* free-radical polymerization. The chitosan nanofibers established hydrogen and covalent bonds with polyacrylamide, and acted as a multifunctional cross-linker and a reinforcing agent in the hydrogel system. The compression strength and storage modulus of the nanocomposite hydrogels were significantly higher than those of the pure polyacrylamide hydrogels. The 1.5% chitosan nanofibers loading showed the best combined swelling and mechanical properties of the hydrogels.

Chitin nanofibrils were easily formed by the gelation of commercial chitin powders with 1-allyl-3-methylimidazolium bromide followed by the regeneration with methanol, according to Kadokawa *et al.* [92]. By using poly(vinyl alcohol) prior to methanol, nanofibrils ~20–60 nm in width and several hundred nanometers in length were obtained. The key point of this facile preparation is that chitin was swollen with said ionic liquid at room temperature, followed by heating at 100 °C (Table 1). Then, a solution of PVA (1.25 mmol, 0.0750 g) in hot water (3.0 mL) was mixed to the gel at 80 °C with stirring; methanol (40 mL) was slowly added to the resulting mixture, and the system was left standing at room temperature for 24 h, followed by sonication to give a dispersion of chitin nanofibrils. Filtration of the dispersion was carried out to give a chitin film.

Hua D.B. *et al.* [96] reported the conjugation of hydrophobic drugs with chitosan, via Schiff reaction in an ionic liquid, which renders chitosan soluble in common organic solvents and amenable to further functional modifications. For example, thermo-responsive poly(*N*-isopropylacrylamide) was grafted to the chitosan-drug conjugate. The graft copolymer self-assembled in water at neutral pH into core-shell nanocarriers with size distribution ~142 ± 60 nm, favorable for intravenous administration. At 37 °C and pH 4.5 (conditions mimicking endosomal or lysosomal uptake) the nanocarriers formed reversed micelles (~8 ± 3 nm) favoring clearance by renal filtration, and 70% of the drug was liberated within 30 h through hydrolytic cleavage of the Schiff base conjugation. Based on the smart drug release profile said approach was deemed viable for the intravenous administration of hydrophobic drugs carried by chitosan-based vehicles.

1-Butyl-3-methyl-imidazolium chloride was used for auto-templating assembly of CaCO_3_ and chitosan to produce well-defined hollow inorganic-organic nanoboxes and nanoframes. By varying the experimental conditions, size and shell-thickness of hollow nanostructures were adjusted in the ranges 200–400 nm and 15–75 nm, respectively [97].

The hybrid film of gold nanoparticles + ionic liquid + chitosan was used as an efficient immobilization matrix to fabricate an immunosensor. The film produced a well-defined voltammetric signal due to the synergistic effects of the ionic liquid and gold nanoparticles. By immobilizing an alkaline phosphatase-labeled antibody in said film, a sensitive amperometric immunosensor was developed for the prostate specific antigen (PSA). Under the optimized conditions, the immunosensor exhibited a linear range from 1.0 to 80 ng/mL of PSA [98]. In a similar fashion, Safavi *et al.* [99] prepared biosensors based on the electrocatalysed reduction or hydrogen peroxide at the electrode coated with cholesterol oxidase. The biosensor exhibited two wide linear ranges of response to cholesterol for concentrations of 0.05–6.2 and 6.2–11.2 mM. The sensitivity was 90.7 μA·mM^−1^·cm^−2^, the limit of detection was 101 μM of cholesterol, and the response time was <7 s.

Porous chitin-based materials were developed by Silva *et al.* [100] who combined the processing of chitin using ionic liquids together with the use of supercritical fluid technique, to provide a clean technology. Chitin was dissolved in 1-butyl-3-imidazolium acetate, followed by regeneration of the polymer in ethanol in specific moulds. The ionic liquid was removed using Soxhlet extraction and successive steps of extraction with supercritical fluid process using carbon dioxide/ethanol ratios of 50/50 and 70/30. Ultralight porous chitin structures were produced while the efficiency of the ionic liquid removal was measured by conductivity. The developed chitin matrices showed interesting features, such as: (i) low crystallinity resulting from loose hydrogen bonds in the chitin structure; (ii) wide mesoporous distribution; (iii) very low density (0.039–0.063 g/L); (iv) porosity between 84 and 90%, and (v) extremely low cytotoxicity for L929 fibroblasts.

## 5. Supercritical Drying

The popularity of supercritical carbon dioxide(sc-CO_2_) stems from the fact that it is nontoxic, nonflammable, promptly available in large amounts, and that it is the second least expensive solvent after water. A special issue devoted to sc-CO_2_ has been published in 2011 by the Journal of Supercritical Fluids. An interesting property associated with the critical state is that the density of the liquid and of the vapor becomes identical, and for this reason the interface between them disappears. Supercritical carbon dioxide is most widely used thanks to its easily accessible critical temperature and pressure (31.2 °C; 7.4 MPa). Supercritical fluid technology is a relatively new technique to obtain micro- and nano-particles: many drugs can be dissolved or liquefied in sc-CO_2_ before being sprayed through a nozzle upon depressurization to produce fine drug particles. It is possible to take advantage from high supersaturation of drugs in sc-CO_2_, which contributes to the particle size reduction; as an alternative, sc-CO_2_ can be used as an antisolvent for the precipitation of drugs from organic solutions [101]. For polar and high MW polymers, the sc-CO_2_ solvent power is unfortunately low, and the use of amphiphiles might be necessary: surfactants, ligands and phase transfer agents should be soluble in sc-CO_2_ to assist in the dissolution process of polymers. Fluorinated polyacrylates, polyethers, and siloxane-based polymers are considered to be sc-CO_2_-philic. The sc-fluid drying processes for preparing protein or polysaccharide-containing powders based on these concepts are described in detail by Jovanovic *et al.* [102]. Diez-Municio *et al.* [103] have investigated the impregnation of chitosan with lactulose using supercritical fluids under various operating conditions, in order to improve the solubility of this natural polymer at neutral or basic pH. The highest impregnation yield (8.6%) was obtained for chitosan scaffolds using the following parameters: continuous process, 60 min contact, 14% (v/v) of co-solvent ethanol:water (95:5), depressurization rate 3.3 bar/min, pressure 100 bar, and 100 °C. Under these conditions, the Maillard reaction took place as well. Biodegradable and mucoadhesive PLGA/chitosan microparticles were manufactured by Casettari *et al.* [104] by using scCO_2_ with the addition of mPEG and chitosan in the absence of organic solvents, surfactants and crosslinkers. Analytical surface techniques, along with the interaction with mucin, demonstrated the presence of the chitosan (<100 μm) on the surface of the particles.

Chitosan solutions were prepared with acetic acid solution, poured in steel containers and frozen at −20 °C to obtain a hydrogel that was treated in different manners: (1) it was dried with air at 40 °C for 10 h; (2) it was put in a bath of acetone at ambient temperature for 24 h to allow the substitution of water with acetone and was dried with air at 40 °C for 8 h; (3) it was put in a bath of acetone at ambient temperature for 24 h and was dried by sc-CO_2_; (4) it was put in a bath of acetone at −20 °C for 24 h and was dried by sc-CO_2_ in the high-pressure vessel filled from the bottom with sc-CO_2_. When the required pressure and temperature were obtained (200 bar and 35 °C), drying was performed for 4 h with the sc-CO_2_ flow rate of about 1 kg/h, that corresponds to a residence time inside the vessel of about 4 min. The depressurization time of 20 min was allocated to bring back the system at atmospheric pressure [105].

**Figure 3:**
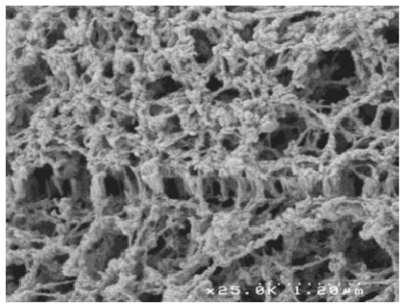
The chitosan fibers are replicated by titanium oxide: the SEM image shows the combined chitosan + titania fibers. Reprinted from [107]. Copyright (2011) with permission from Elsevier.

When the preference was given to low temperature water substitution followed by supercritical gel drying to prevent the collapse of the chitosan gel, water substitution with acetone was performed at the same temperature of the gel formation (−20 °C for 24 h). Subsequently, sc-CO_2_ gel drying was performed at the same processing conditions. In this case, the 3-D shape and the size of samples were preserved, as shown in Figure 3.

The obtained structures present a morphology very similar to the extracellular matrix, *i.e.*, a finely interconnected nano sub-structure, and therefore they can be suitable scaffolds. In fact, this kind of nanometric fibrous network is the ideal environment for cell adhesion and growth for the regeneration of cartilages, skin and bone [105]. The surface properties of biological materials are at the basis of phenomena like cell adhesion, formation of bacterial films, and recognition between biological units. Surface polarity is a major parameter in controlling the adhesion of cells and bacteria, a relevant phenomenon in fields as different as safety of surgical devices, colonization of biomaterials, and sensitivity of biological sensors.

Based on these considerations, supercritically dried aerogels of several polysaccharides have been characterized by Robitzer *et al.* [106]. The nature of the functional groups of the polysaccharide significantly influences the adsorption of N_2_ on the surface of the aerogel. Surface area values as high as 570 m^2^g^−1^ have been measured. The net enthalpy of adsorption increases with the polarity of the chemical groups of the polymer, in the order: chitin < agar ≤ chitosan < carrageenan < alginic acid/alginate. The surface area and the mesopore distribution of the aerogels depend on the dispersion of the parent hydrogel and on the behavior of each polymer during the drying treatment. Aerogels retaining the dispersion of the parent hydrogel are mainly macroporous (pores larger than 50 nm) while materials liable to shrink upon solvent exchange form mesoporous structures.

**Figure 4:**
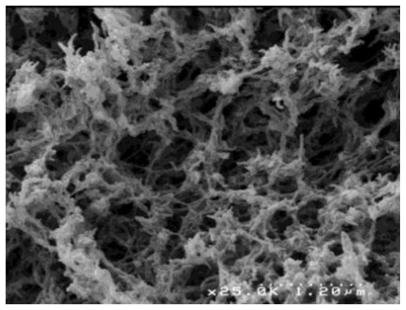
Removal of chitosan leads to pure titanium oxide with filamentous structure. Reprinted from [107]. Copyright (2011) with permission from Elsevier.

El Kadib *et al.* [107] described a versatile strategy for fabricating highly porous and nanofibrous titania, zirconia, alumina and tin oxide. By taking advantage from the favorable effect of scCO_2_ drying to avoid the collapse of the transient hybrid material network, all targeted metal oxides were produced, after calcination, as fibrous filaments featuring dual meso- and macro-porous network with surface area ranging from 110 to 310 m^2^g^−1^, as shown in Figures 3 and 4.

As far as silica is concerned, hydrophobic chitosan+silica hydrogels were prepared by Ayers *et al.* [108]: dried aerogels were exposed to hexamethyldisilazane vapors at 60 °C. After supercritical drying, uncracked monoliths with very little shrinkage were obtained. When exposed to water, said aerogels adsorbed a small amount of liquid at their outer surface, while maintaining their shape. The Brunauer–Emmett–Teller (BET) surface area of said aerogels was very large, 472–750 m^2^/g, depending on the ratio chitosan/silica.

In a more elaborated preparation, a solution of chitosan (1%) was mixed with the crosslinker genipin solution (4%) [17]: stirring was continued until the solution turned into a viscous gel. Then the hydrogel was subjected to solvent exchange into acetone thrice to remove water from the structure. After solvent exchange, the chitosan–genipin derivative was placed inside a sealed chamber of the sc-CO_2_ extractor at 40 °C and 200 bar. The reaction was left for 2 h and a flow of CO_2_ was then applied through the sample in order to replace all the organic solvent with CO_2_. The pressure was then released slowly to the atmosphere and the temperature was reduced to 20 °C. The chitosan derivative had BET surface area of 49 m^2^/g, with a monolayer volume of 11 cm^3^/g. The porosimetry result showed that genipin-crosslinked chitosan scaffold had adequate surface area to provide cell adhesion and proliferation. In fact the osteoblast proliferation on chitosan–genipin scaffolds was assessed using Almar Blue assay and found to be satisfactory considering that the number of cells attached to the genipin-crosslinked chitosan scaffolds after 1, 3, and 7 days of cell culture increased with time thus indicating the suitability of the material for tissue engineering applications [109,110].

The surface of chitin too can be expanded to large values: rapid expansion techniques with sc-CO_2_ were used by Salinas-Hernandez *et al.* [111] to form chitin microstructures. Depending on the experimental conditions, they obtained either spherical microparticles with diameters 1.7–5.3 μm with the rapid expansion of sc-solution technique, or continuous microfibers with diameters 11.5–19.3 μm with the rapid expansion into water technique, possibly influenced, in the latter case, by the high forces in the hydrogen bond network of the molecular structure of chitin. It is important to mention that the present method makes it possible to produce much more uniform and thinner nanofibers on a larger scale than the electrospinning process, without using any hazardous chemicals.

## 6. Conclusion

This bibliographic survey shows the high viability of basic chemical and physical sciences that are promoting the applicability of chitin and chitosan in a number of demanding areas. Just in the most recent years a real interface of existing technologies and chitin science has taken place, and moreover new technologies are emerging in a few scattered articles (hot melt blending, processing, extrusion and stretching [112–115]; foaming [116]; electrofiltration [117]; decrystallization [118]; sonolysis, microfluidization and shearing [119]) that will certainly contribute to the exploitation of renewable chitin-bearing resources.

## Figures and Tables

**Table 1:** Solubility of isolated chitins and chitosan in 4 ionic liquids. Based on data in [91,92].

Polymer	Origin and viscosity	Solubility (w/w%) at 110 °C			

		AmiCl	AmiBr	BmiAc	BmiCl
		
α-Chitin	Crab	n.a.	Soluble, 9.1	n.a	n.a.
α-Chitin	Crab, η 35 cp	Insoluble	n.a.	Soluble, 6	Partly soluble
β-Chitin	Squid pen, η 15 cp	Insoluble	n.a.	Soluble, 7	Partly soluble
β-Chitin	Squid pen, η 278 cp	Insoluble	n.a.	Soluble, 3	Insoluble
Chitosan	Crab, Mv 97 kDa	Soluble, 8	n.a.	Soluble, 12	Soluble, 10

AmiCl is 1-allyl-3-methylimidazolium chloride; BmiCl is 1-butyl-3-methylimidazolium chloride, BmiAc is 1-butyl-3-methylimidazolium acetate, and AmiBr is 1-allyl-3-methylimidazolium bromide. n.a. = not available.
